# Comparison of PET tracing and biodistribution between ^64^Cu-labeled micro-and nano-polystyrene in a murine inhalation model

**DOI:** 10.1186/s12989-023-00561-7

**Published:** 2024-01-31

**Authors:** Joycie Shanmugiah, Javeria Zaheer, Changkeun Im, Choong Mo Kang, Jin Su Kim

**Affiliations:** 1 Division of RI Application, Korea Institute of Radiological and Medical Sciences, 75 Nowon-Gil, Gongneung-Dong, Nowon-Gu, Seoul, 01812 Korea; 2 Radiological and Medico-Oncological Sciences, Korea National University of Science and Technology, Seoul, 01812 Republic of Korea

**Keywords:** Micro-polystyrene, Nano-polystyrene, ^64^Cu, PET, Inhalation, Plastic

## Abstract

**Introduction:**

Recent studies showed the presence of microplastic in human lungs. There remains an unmet need to identify the biodistribution of microplastic after inhalation. In this study, we traced the biodistribution of inhaled micro-sized polystyrene (mPS) and/or nano-sized PS (nPS) using ^64^Cu with PET in mice.

**Methods:**

We used 0.2–0.3-µm sized mPS and 20-nm sized nPS throughout. ^64^Cu-DOTA-mPS, ^64^Cu-DOTA-nPS and/or ^64^CuCl_2_ were used to trace the distribution in the murine inhalation model. PET images were acquired using an INVEON PET scanner at 1, 12, 24, 48, and 72 h after intratracheal instillation, and the SUV_max_ for interesting organs were determined, biodistribution was then determined in terms of percentage injected dose/gram of tissue (%ID/g). Ex vivo tissue-radio thin-layer chromatography (Ex vivo-radioTLC) was used to demonstrate the existence of ^64^Cu-DOTA-PS in tissue.

**Results:**

PET image demonstrated that the amount of ^64^Cu-DOTA-mPS retained within the lung was significantly higher than ^64^Cu-DOTA-nPS until 72 h; SUV_max_ values of ^64^Cu-DOTA-mPS in lungs was 11.7 ± 5.0, 48.3 ± 6.2, 65.5 ± 2.3, 42.2 ± 13.1, and 13.2 ± 2.3 at 1, 12, 24, 48, and 72 h respectively whereas it was 31.2 ± 3.1, 17.3 ± 5.9, 10.0 ± 3.4, 8.1 ± 2.4 and 8.9 ± 3.6 for ^64^Cu-DOTA-nPS at the corresponding timepoints. The biodistribution data supported the PET data with a similar pattern of clearance of the radioactivity from the lung. nPS cleared rapidly post instillation in comparison to mPS within the lungs. Higher accumulation of %ID/g for nPS (roughly 2 times) were observed compared to mPS in spleen, liver, intestine, thymus, kidney, brain, salivary gland, ovary, and urinary bladder. Ex vivo-radioTLC was used to demonstrate that the detected gamma rays originated from ^64^Cu-DOTA-mPS or nPS.

**Conclusion:**

PET image demonstrated the differences in accumulations of mPS and/or nPS between lungs and other interesting organs. The information provided may be used as the basis for future studies on the toxicity of mPS and/or nPS.

**Graphical abstract:**

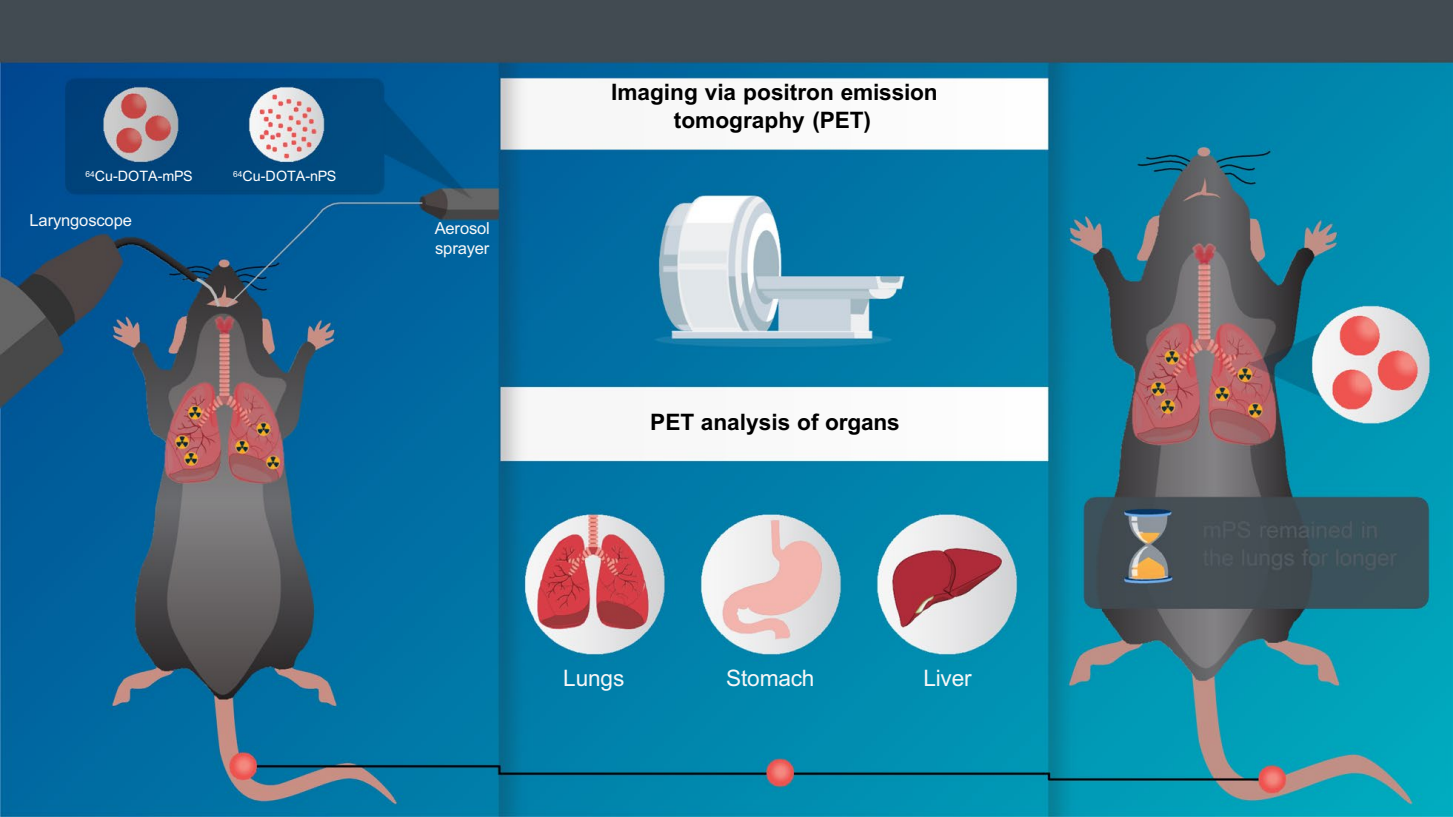

**Supplementary Information:**

The online version contains supplementary material available at 10.1186/s12989-023-00561-7.

## Introduction

Microplastics were subsequently found to accumulate in aquatic ecosystems and marine life [1, 2] and in the deep organs of humans [3], contributing significantly to environmental and public health concerns [4]. Living beings are constantly prone to exposure to microplastic by ingestion, and/or inhalation [5]. Several studies in which microplastic fed mouse models developed various physiological variations as well as genetic-level mutations, resulting in disorders [6–9]. Polystyrene (PS) is one of the widely used plastic for most daily-use products and we traced the biodistribution of orally administered ^64^Cu-labeled PS in mice by PET [10].

Nanoplastics with dimensions ranging from 1 to 100 nm have been observed to show adverse effects on the environment and human health [11–13]. Mechanical stress, ultraviolet radiation, and chemical degradation can result in the fragmentation of microplastic to nanoplastic. Since the size of nano-plastics are lesser than 100 nm diameter, there is high possibility that they can enter cells and disrupt the cellular activity. Hence it is important to look into the potential toxicity of nanoplastics via inhalation as well [14].

Inhalation was identified as an exposure route for microplastic when recent studies revealed deposition of microplastics even in human lungs [3, 15]. While microplastic evidently enter the human body through diet [16–18], airborne microplastic present indoors and outdoors may also be a dominant source [19–21]. The potential harmful effects of intratracheally instilled 5-µm microplastics were reported in the lungs of mice [22].

**Fig. 1:**
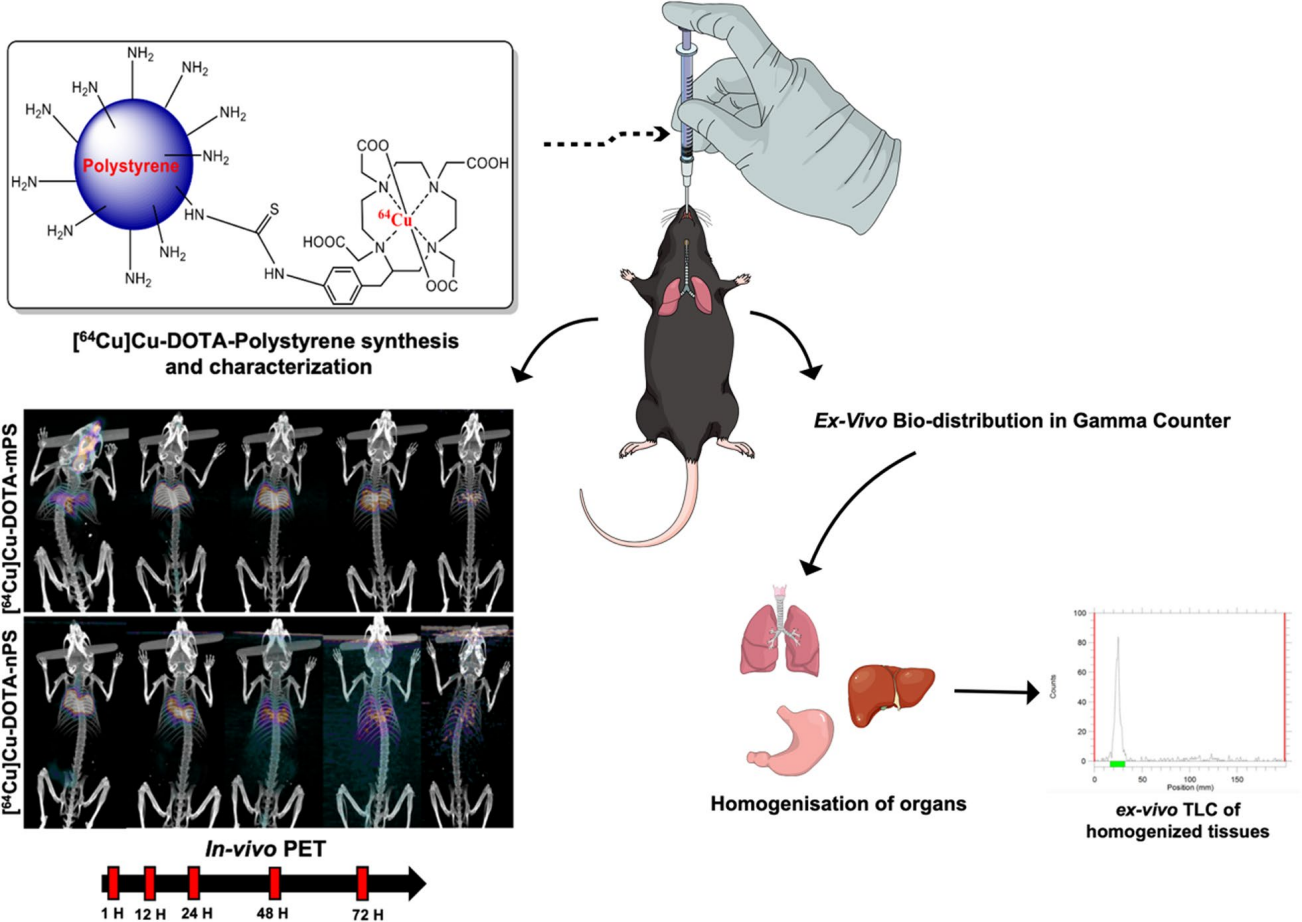
Schematic of the experiment. ^64^Cu-DOTA-mPS/nPS was synthesized and characterized using HPLC and radiochemical purity was analyzed using radio-TLC. ^64^Cu-DOTA-mPS/nPS was intra-tracheally instilled into mice and PET imaging was performed. The organs were extracted at each timepoint and radioactivity was counted in a gamma counter followed by tissue homogenization and ex-vivo TLC analysis to confirm the stability of the labelled products in each organ (mPS = micro polystyrene, nPS = nano polystyrene)

Until now, the translocation of microplastics such as PS from the pulmonary tissue to other organs has not been thoroughly explored. In addition, the difference of biodistribution during inhalation between micro-sized PS (mPS) and nano-sized PS (nPS) remains unclear. Figure 1 shows the schematic figure of this study. To visualize the distribution of mPS and nPS in the respiratory system, the PS were labeled with the PET radioisotope ^64^Cu and instilled directly into the trachea of mice. An aerosol sprayer was used to ensure that ^64^Cu labeled PS was distributed equally in the lungs. Then, we compared the biodistribution of PS in the respiratory system and their subsequent distribution to other parts of the body post-instillation using ^64^Cu labeled mPS and/or nPS in vivo PET*,* and ex vivo biodistribution for this study.

## Materials and methods

### Conjugation and ^64^Cu labeling PS

Surface-modified mPS with particle size between 0.2–0.3 μm was obtained commercially (Spherotech™ amino-polystyrene particles; Lake Forest, IL, USA) and conjugated with S-2-(-4-isothiocyantobenzyl)-1,4,7,10 tetraazacyclododecane tetraacetic acid (p-SCN-Bn-DOTA; Macrocyclics™) as previously reported by our research group [10]. Briefly, 2 mg of mPS was added to 240 µL of 0.1 M sodium carbonate buffer (pH 9.0) along with 770 µg of p-SCN-Bn-DOTA in 100 µL of deionized water (Sigma-Aldrich) and incubated at 25 °C with mild shaking in a thermomixer at 1000 rpm for 20 h. The incubated mixture was purified using a Vivispin 20 centrifugal concentrator (Sartorius Stedium Biotech, USA) with a 100-kDa cut-off.

The above-mentioned procedure was also used for radiolabeling nPS with 20 nm amino-polystyrene purchased from Nanocs Inc. (New York, NY, USA). nPS was washed with deionized water containing 0.05% Tween-20 (Sigma-Aldrich, Saint Louis, MO, USA) with centrifugation in a Vivispin 20 centrifugal concentrator (10-kDa molecular weight cutoff [MWCO], Sartorius Stedium Biotech, USA) to prevent aggregation, and sonicated for 10 min before conjugation with p-SCN-Bn-DOTA. The chelator-conjugated nPS was purified in a Vivispin 20 centrifugal concentrator (10 kDa), and the final concentrations of both mPS and nPS after conjugation with p-SCN-Bn-DOTA was 2 mg/100 µL. The number of moles of DOTA conjugated per milligram of the PS beads was determined using high-performance liquid chromatography (HPLC), and characterization of DOTA-PS was performed using the SU8010 ultra-high-resolution field emission scanning electron microscopy (FE-SEM) system.

^64^Cu produced in the cyclotron (installed in KIRAMS) was dried by argon gas purging and re-dissolved in 0.01 N HCL (9.25 MBq/µL). To the conjugated mixture containing DOTA-PS, 40.7 MBq of ^64^CuCl_2_ was added and incubated at 40 °C for 30 min at 1000 rpm along with 80 µL of sodium acetate buffer (pH 5.0). The radiolabeled mPS was purified using a 100-kDa MWCO centrifugal filter for 30 min at 3500 rpm. This step was repeated twice with deionized water until the final volume was reduced to less than 200 µL. On the other hand, radiolabeled nPS was incubated with 50 mM ethylenediaminetetraacetic acid (EDTA, pH 6.0) for 15 min prior to purification using a 10-kDa MWCO centrifugal filter for 30 min at 3500 rpm and 25 °C. EDTA, which is a strong chelator, binds to free copper [23], ensuring better purification and greater radiochemical purity. The ^64^Cu-labeled-mPS and nPS were then diluted with phosphate-buffered saline depending on the appropriate volume of instillate (3 µL per gram of mouse weight[24]). The radiochemical purity of the labeled product was determined by instant thin-layer chromatography with citric acid (0.1 M) in water as the mobile phase.

### In vitro stability

An in vitro stability test was conducted for ^64^Cu-DOTA-mPS and ^64^Cu-DOTA-nPS by adding 3.08 MBq in 50 µL to 200 µL of mouse serum, simulated lung fluid (Biochemazone™), and phosphate-buffered saline (PBS). The samples were incubated at 37 °C for 72 h (until the end of the study period). At each time point, instant thin-layer chromatography (TLC) was performed with 0.1 M citric acid in water as the mobile phase to determine the percentage stability.

### Animal preparation and intratracheal instillation

All animal experiments were performed in accordance with the institutional guidelines of the Korea Institute of Radiology and Medical Science (IACUC number: KIRAMS 2022–0079). C57BL/6 J mice (n = 80; female, 8 weeks old; Shizuoka Laboratory Center, Japan) were used for the study and were allowed food pellets and water ad libitum during the entire course of the study. The mice were anesthetized via an intraperitoneal cocktail injection of ketamine hydrochloride (50–80 mg/kg body weight) and xylazine (8–12 mg/kg body weight). Once adequate anesthesia was observed by paw pinch stimulus, each mouse was fixed with its incisors to a rubber band on a board inclined at an angle of 45° to the lab bench. A mini laryngoscope (Hillrom™, USA) was used to clearly visualize the tracheal opening along with curved blunt-ended forceps to grasp the tongue, and the radioactive substance (after vortexing) was loaded into a 1-mL insulin syringe and gently instilled as an aerosol in the trachea by using an aerosol sprayer (Natsume Seisakusho, Tokyo, Japan) attached to the syringe. Post-instillation, the mice were monitored for breathing difficulty.

### PET/CT imaging

Mice (n = 5 per group) received intratracheal instillation of ^64^Cu-DOTA-mPS and/or nPS (1.1 MBq in 50 µL) or ^64^CuCl_2_ (1.1 MBq in 50 µL) for imaging. PET images were acquired 1, 12, 24, 48, and 72 h post-instillation. PET/CT image acquisition was performed on a Siemens Inveon PET Scanner (Siemens Medical Solutions, Germany) for 15 min per acquisition, with an energy window of 350–650 keV. Reconstruction of the acquired PET images was performed using the SP-MAP algorithm. The matrix size was 128 × 128 × 159, and the voxel size was 0.776 × 0.776 × 0.796 mm^3^.

For the acquisition of anatomical images, CT imaging was acquired with the second bed position, full rotation, and 180 projections per bed position. The exposure time was 200 ms, and the estimated scan time was 504 s. CT data were reconstructed using Feldkamp reconstruction with a Shepp–Logan filter. The effective pixel size of the reconstructed CT images was 109.69 μm × 109.69 μm. PET and CT images were co-registered using the Inveon Research Workplace (Siemens Medical Solutions Inc.).

### Standard uptake value

Region-of-interest analysis (ROI) to determine the uptake of the instilled tracer in major organs and tissues was performed using Inveon Research Workplace software (Siemens Medical Solutions Inc.). After co-registration of CT and PET data, the ROI was drawn on the CT image and copied to the PET data. A nuclear medicine expert drew the ROI after identifying the location in three cross-sectional images for precise ROI of the lungs, stomach, and liver (Additional file 3: Fig. S3-Additional file 5: Fig. S5). The maximal value within the ROI was used to avoid partial volume effects for the calculation of the standard uptake value (SUV).

### Biodistribution of ^64^Cu-DOTA-polystyrene instilled mice

Biodistribution was evaluated at 1, 12, 24, 48, and 72 h after intratracheal instillation of ^64^Cu-DOTA-mPS and/or nPS (n = 5 at each time point) or ^64^CuCl_2_ (n = 3 at each time point). Mice were euthanized, and major organs and blood were collected *in toto* while avoiding cross-contamination. Radioactivity was measured as counts per minute (CPM) in a WIZARDS2 Automatic γ-counter (PerkinElmer) and recorded in terms of percentage injected dose per gram of tissue (%ID/g).

### Ex vivo tissue-radio thin-layer chromatography (Ex vivo-radioTLC)

To evaluate whether the counts recorded from the sample at 1 h post-instillation were from ^64^Cu-DOTA-mPS, nPS or ^64^Cu, ex vivo tissue-radio TLC was performed with homogenized samples collected 1 h post-instillation. The tissues were homogenized using 10% SDS-PBS (pH 7.4), as described previously [10].

### Statistical analysis

The quantitative data obtained were expressed as mean $\pm$ SD. Student’s *t*-test was used to compare the means using PRISM software (GraphPad ver. 5.0, San Diego, CA, USA), and *P* values less than 0.05 were considered statistically significant.

## Results

### Chelation and radiolabeling

After radiolabeling, the purified ^64^Cu-DOTA-PS had a radiochemical purity of 95.56%$\pm$ 0.31% for mPS and 94.21%$\pm$ 0.33% for nPS, as determined by ex vivo radio instant thin layer chromatography (Additional file 1: Fig. S1A), and the radiolabeling yield was 92.58% $\pm$ 0.7% and 50.67% $\pm$ 0.1%, respectively.

**Fig. 2:**
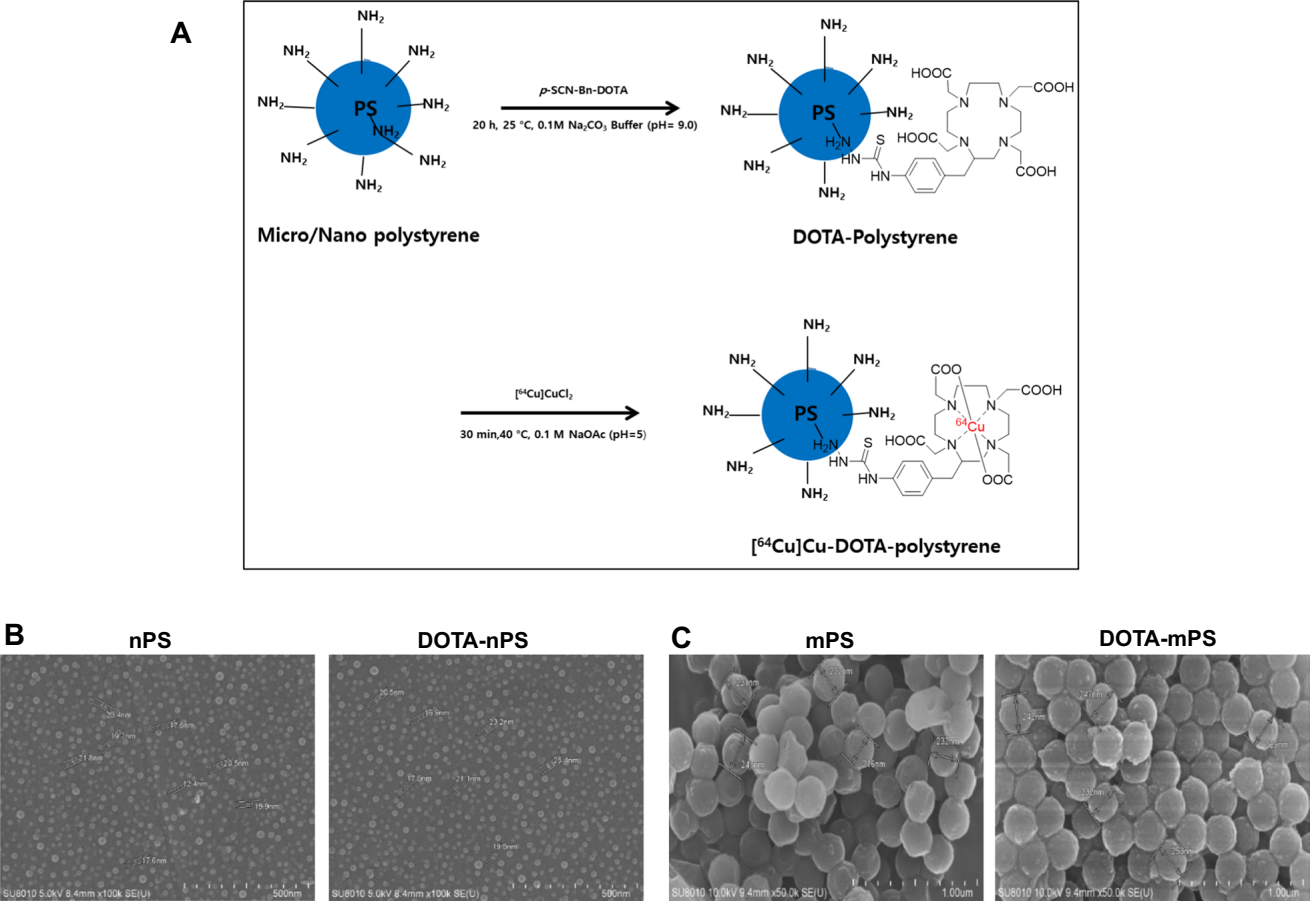
Radiolabelling of.^64^Cu-DOTA-polystyrene and characterization of DOTA-mPS and DOTA-nPS using field emission scanning electron microscope. (**A**) Conjugation of DOTA with polystyrene under specific conditions. (**B**) Field emission scanning electron microscope results show no difference between polystyrene bead and DOTA-polystyrene bead of both sizes (mPS = micro-polystyrene, nPS = nano-polystyrene)

DOTA conjugation was quantified using high-performance liquid chromatography (HPLC), and 690 nmol and 630 nmol of DOTA were conjugated per milligram of mPS and nPS, respectively (Additional file 2: Fig. S2A). The scanning electron microscopy (SEM) data indicated that the diameter of polystyrene after DOTA conjugation was 237.6 nm ± 5.1 nm for mPS and 21.6 nm ± 1.4 nm for nPS, indicating negligible differences from the amino-polystyrene size prior to conjugation (Fig. 2).

### In vitro stability

The results of the in vitro stability test indicated that the labeled product was stable over the entire period of the study. The percentage stability of ^64^Cu-DOTA-mPS and ^64^Cu-DOTA-nPS in PBS (pH 7.4), simulated lung fluid, and mouse serum was shown in Additional file 1: Fig. S1B.

### Ex vivo tissue-radio thin-layer chromatography

The *ex-vivo* iTLC performed for lungs, stomach and liver at 1 h confirmed that the counts from the organs belong to ^64^Cu-DOTA-mPS/nPS and not ^64^Cu proving that the labelled product is stable *in-vivo*. The results are shown in Additional file 2: Fig. S2B. It was ensured that there was no de-chelation of the radio-labelled PS in the stomach due to the acidic environment by *ex-vivo* instant thin-layer chromatography (iTLC) and the results confirmed that the ^64^Cu-DOTA-mPS/nPS was intact at 1 h. The *ex-vivo* iTLC results shown in Additional file 2: Fig. S2B explains that the radioactivity emanating from the organs are from the ^64^Cu-DOTA-mPS/nPS and not from ^64^Cu since the labelled product remains at origin and ^64^Cu travels to the solvent front in TLC when 0.1 M citric acid is used as mobile phase[25].

### PET imaging

**Fig. 3:**
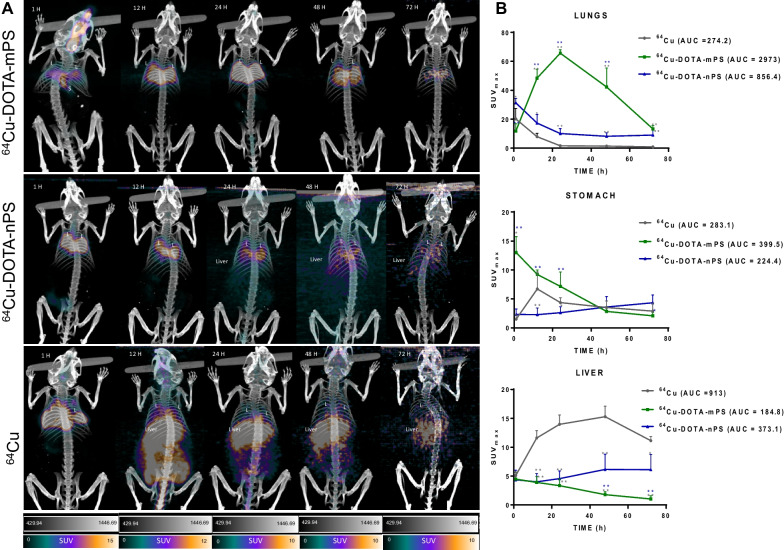
(**A**) Representative PET-CT images of intra-tracheally instilled ^64^Cu-DOTA-mPS, ^64^Cu-DOTA-nPS and ^64^Cu at all time points displaying retention of polystyrene in lungs compared to ^64^Cu that is rapidly cleared from the lungs post instillation. (**B**) The graphs represent SUV_max_ for important organs such as lungs, stomach and liver along with the AUC which is maximum for mPS in lungs and ^64^Cu in liver. AUC = area under curve; mPS = micro-polystyrene; nPS = nano-polystyrene; L = lungs; S = stomach (*P < 0.05, **P < 0.005, * significance in comparison to ^64^Cu; * significance in comparison to.^64^Cu-DOTA-nPS)

PET imaging was performed until 72 h post-intratracheal instillation of ^64^Cu-DOTA-mPS and/or nPS or ^64^CuCl_2_ along with a biodistribution study at the set time points. The acquired sequential PET/CT images are presented in Fig. 3A and Additional file 3: Fig. S3. Quantitative data obtained in terms of SUV_max_ for the lungs, stomach, and liver were plotted against time (Fig. 3B).

The PET images clearly indicated the presence of ^64^Cu-DOTA-mPS within the lungs (marked “L” in Fig. 3A) for up to 72 h, with an SUV_max_ of 11.7 $\pm$ 5.0, 48.3 $\pm$ 6.2, 65.5 $\pm$ 2.3, 42.2 $\pm$ 13.1, and 13.2 $\pm$ 2.3 at 1, 12, 24, 48, and 72 h, respectively. These values were significantly different from those for ^64^CuCl_2_ and ^64^Cu-DOTA-nPS at all time points after 12 h. The AUC-lung for ^64^Cu-DOTA-mPS was 10.84-fold and 3.47-fold that of ^64^Cu and ^64^Cu-DOTA-nPS, respectively (Fig. 3B).

However, slight transit of the radiolabeled mPS from the instilled location in the trachea into the stomach was observed in the initial 1 h image, which then decreased substantially in the later images, with SUV_max_ values of 13.0 $\pm$ 2.7, 9.2 $\pm$ 0.7, 7.1 $\pm$ 2.5, 2.8 $\pm$ 0.6, and 2.0 $\pm$ 0.5 at 1, 12, 24, 48, and 72 h, respectively, in the stomach. Furthermore, radioactivity was observed in the liver, which decreased gradually over time with SUV_max_ values of 4.4 $\pm$ 1.1, 3.9 $\pm$ 1.0, 3.3 $\pm$ 1.2, 1.7 $\pm$ 0.5, and 1.0 $\pm$ 0.5 at 1, 12, 24, 48, and 72 h, respectively.

The PET of ^64^Cu-DOTA-nPS exhibited a different pattern, with maximum activity and a markedly elevated SUV_max_ observed in the lung (marked “L” in Fig. 3A) post-instillation. The lungs showed SUV_max_ values of 31.2 ± 3.1, 17.3 ± 5.9, 10.0 ± 3.4, 8.1 ± 2.4, and 8.9 ± 3.6 at 1, 12, 24, 48, and 72 h, respectively. Although the radioactivity observed in the stomach was not as high as that in the ^64^Cu-mPS group, some ^64^Cu-DOTA-nPS radioactivity was observed in the stomach, with SUV_max_ values of 2.3 ± 0.9, 2.2 ± 1.1, 2.6 ± 1.0, 3.6 ± 1.8, and 4.3 ± 1.3 at 1, 12, 24, 48, and 72 h, respectively. The liver accumulated the radiolabeled nPS gradually post-instillation, with an SUV_max_ of 6.1 ± 2.6 at 72 h.

In the ^64^Cu-instilled mice, radioactivity was distributed instantly into the entire lungs after intratracheal instillation, and less uptake was detected in the stomach, unlike in the radiolabeled mPS-instilled group. However, activity in the lungs was translocated relatively early. The SUV_max_ in the lungs was 20.1 $\pm$ 7.2 at 1 h post-instillation, after which it decreased rapidly to 7.8 $\pm$ 2.2 at 12 h, 1.6 $\pm$ 0.5 at 24 h, 1.4 $\pm$ 0.1 at 48 h, and 0.85 $\pm$ 0.20 at 72 h. ^64^Cu activity, however, accumulated in the liver (marked “Liver” in Fig. 3A) in the initial image at 1 h, possibly as a result of ^64^Cu crossing the air–blood barrier (ABB) into the blood circulation and sequestering by the liver [25]. The SUV_max_ values in the liver were 5.1 $\pm$ 0.6, 11.6 $\pm$ 1.2, 13.9 $\pm$ 1.6, 15.2 $\pm$ 1.8, and 11.1 $\pm$ 0.7 at 1, 12, 24, 48, and 72 h, respectively (Fig. 3B, *P < 0.05, **P < 0.005). Moreover, the AUC-liver was 4.94-fold and 2.44-fold that for ^64^Cu-DOTA-mPS and ^64^Cu-DOTA-nPS, respectively. Additionally, observable uptake was noted in the small intestine, large intestine, kidney, and urinary bladder.

### Biodistribution

**Fig. 4:**
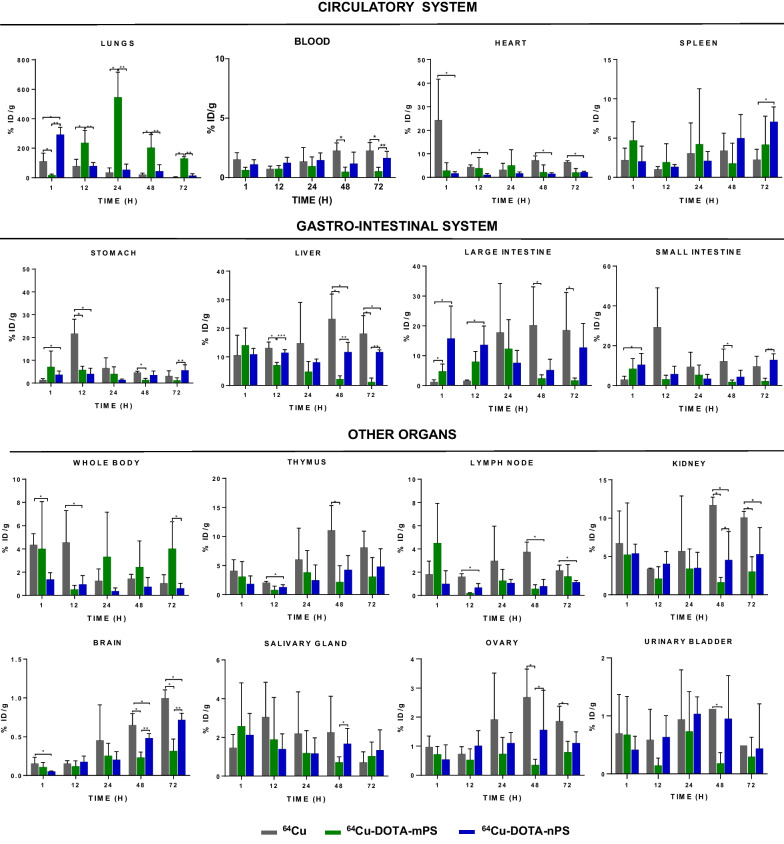
Bio-distribution data of the intra-tracheally instilled ^64^Cu-DOTA-mPS (n = 5), ^64^Cu-DOTA-nPS (n = 5) and ^64^Cu (n = 3) groups for all organ systems. The instilled radioactivity can be seen distributed to various organs especially stomach, small intestine, large intestine, liver, spleen, kidneys, and thymus. The data suggests that ^64^Cu subsequently crossed the ABB in the lungs and got accumulated in the liver by 12 h and also in other organs in small amounts. However, mPS and nPS retained in the lungs and cleared slowly as compared to ^64^Cu. Faster clearance from the lungs to other organs like liver can be noted in nPS data as compared to the mPS bio-distribution results. The %ID/g in the lung for mPS was significantly different than that of ^64^Cu and nPS at all time points. mPS = micro-polystyrene; nPS = nano-polystyrene. (*P < 0.05, **P < 0.005, ***P < 0.0005 Student *t* test)

Interestingly, the distribution pattern from the biodistribution data (Fig. 4) was similar to that observed in the PET images. The γ-counting data were consistent with the PET/CT scan data, and the mean %ID/g of ^64^Cu-DOTA-mPS in the lungs was significantly higher than that of ^64^Cu and ^64^Cu-DOTA-nPS at 72 h post-instillation (Fig. 4, *P < 0.05, **P < 0.005).

The lung retention of ^64^Cu-DOTA-nPS was lower than that of ^64^Cu-DOTA-mPS, but 3.9-fold higher than the mean %ID/g of ^64^Cu at 72 h. However, the mean %ID/g of ^64^Cu-DOTA-nPS in the liver was considerably greater after 72 h, being 9.9-fold that of ^64^Cu-DOTA-mPS.But the %ID/g of ^64^Cu-DOTA-nPS was 1.56-fold lower than the %ID/g of ^64^Cu in the liver at 72 h.

On the other hand, the ^64^Cu-instilled group showed almost complete clearance from lungs (%ID/g 3.6%$\pm$ 3.2% at 72 h; *P < 0.05, **P < 0.005) as well as the highest liver uptake among all the three groups at all time points, which gradually increased post-instillation until 48 h, indicating the translocation of ^64^Cu from the lungs by crossing the ABB. The %ID/g for the liver in the ^64^Cu group was 10.6 $\pm$ 6.9%, 13.1 $\pm$ 2.0%, 14.8 $\pm$ 14.2%, 23.2 $\pm$ 8.7%, and 18.1 $\pm$ 6.2% at 1, 12, 24, 48, and 72 h.

In addition, we identified the uptake of radiolabeled mPS and nPS in the circulatory systems such as blood, heart, spleen, and gastro-intestinal system such as liver, intestine, and the other organs such as thymus, lymph node, kidney, salivary glands, urinary bladder, and even to brain. The mean %ID/g of ^64^Cu-DOTA-nPS in brain was 2.26-fold higher than mPS and %ID/g of ^64^Cu-DOTA-nPS in spleen was 3.12-fold higher than ^64^Cu at 72 h (*P < 0.05, **P < 0.005). By the end-point, blood had increased uptake of ^64^Cu (%ID/g 2.20%$\pm$ 0.68%) followed by nPS (%ID/g 1.60%$\pm$ 0.55%) and then mPS (%ID/g 0.50%$\pm$ 0.33%). Though not significant, the mean %ID/g of ^64^Cu-DOTA-nPS at 72 h was higher than that of mPS in thymus, kidneys, salivary glands, ovaries, and urinary bladder.

## Discussion

To the best of our knowledge, we first revealed the in vivo biodistribution of mPS and nPS after intratracheal instillation in mice using PET and ex vivo biodistribution assays using γ-counter. In our study, PET imaging, and post-mortem organ biodistribution data indicated that ^64^Cu-DOTA-mPS primarily remained within the lungs of mice for up to 72 h after instillation. Although the radioactivity gradually decreased over time, the %ID/g in the lungs at 72 h in mice that received instillation of ^64^Cu-DOTA-mPS was significantly higher than that in mice receiving ^64^Cu. ^64^Cu-DOTA-nPS also showed retention of radioactivity within the lungs, but most of the nPS was cleared off rapidly in comparison with the corresponding findings for mPS. We identified higher accumulation of %ID/g for nPS (roughly 2 times) compared to mPS in spleen, liver, small and large intestine, thymus, kidney, brain, salivary gland, ovary, and urinary bladder at 72 h after intratracheal instillation (Fig. 4).

Fast clearance of nPS compared to mPS in lung would be possibly because nPS may easily cross the ABB into the systemic circulation from the alveolar region [26]. Mode of elimination involves the transfer into the pulmonary lymph nodes after phagocytosis by alveolar macrophages [27, 28], which is a prominent clearance mechanism in the alveolar region, especially for insoluble particles. Similarly, ultrafine particles such as PM_0.1_ (particles less than 100 nm in diameter) could enter the bloodstream by dissolving rapidly in the lipid and/or the fluid lining the alveolar wall [29]. Uptake into the bloodstream and lymphatics occurs in the interstitium via diffusion. Fast translocation into the ABB could be explained by following processes: (a) cell-mediated active transportation, (b) passive transportation such as diffusion, and (c) active or passive transportation through the pores in the endothelial cells or gaps between the alveolar epithelial cells [30]. Further, the differences in the rate of clearance can be explained on the basis of the modes of elimination, since larger-sized plastics (mPS in this study) are phagocytosed by alveolar macrophages and eliminated by the muco-ciliary apparatus or remain in the lung interstitium, whereas smaller plastics (nPS in this study) may enter the blood capillaries [30]. The pharmacokinetics of PS may differ depending on the modification properties, such as the size and surface chemistry, and it may affect in vivo behavior once it enters the systemic circulation. Molecular weight and sizes play important roles in the clearance mechanism [31]. Renal and hepatic clearance play important roles in the elimination of nano- and micro-sized particles from the circulatory system [32]. Consistent with these findings, ^64^Cu-DOTA-mPS/nPS were also taken up by the liver or excreted through the renal route. Liver and spleen uptake can be explained as a function of the reticuloendothelial system (RES) to phagocytose mPS and nPS in the systemic circulation. The uptake of nPS in the liver increased, similar to the uptake of ^64^Cu, however, the distribution of ^64^Cu-DOTA-nPS observed in other organs were different in comparison to ^64^Cu. Whereas, the %ID/g of ^64^Cu-DOTA-mPS in the liver was high only at the initial time point and then decreased gradually. Our previous study on the oral administration of ^64^Cu-DOTA-mPS also revealed liver activity [10]. The %ID/g of ^64^Cu-DOTA-mPS and/or nPS in the brain increased gradually post-instillation. The blood–brain barrier (BBB) consists of brain endothelial cells attached to the basement membrane and linked together by tight junctions, and even small-molecule drugs can barely cross the BBB due to these properties [33]. Therefore, the absolute value of ^64^Cu-DOTA-PS brain uptake was less and size-dependent; thus, the nPS concentration was higher than that of mPS. Previously, we reported ingested polyethylene would be as possible risk factor for autism spectrum disorder [8]. According to these findings in this study, nPS may be more likely to damage the brain than mPS, which requires further longitudinal research.

The stomach, liver, and intestine were not clearly visible from Fig. 3A and Additional file 3: Fig. S3 due to large differences of PET intensity. However, after window adjustment, higher uptake in the trachea and lungs were saturated and then PET uptake in stomach, liver, and intestine could be clearly visible (Additional file 4: Fig. S4, Additional file 5: S5). Higher stomach activity was observed in the mPS group immediately post-instillation. Here, PET uptake in the stomach could be explained due to the muco-ciliary clearance mechanism in the respiratory tract. Several studies involving bio-distribution of intra-tracheally instilled radioisotope labelled particles had shown stomach uptake as well [34, 35]. Muco-ciliary clearance was observed when PM_10_ (particles less than 10 μm in diameter) were deposited in the trachea [36]. The muco-ciliary mechanism of the respiratory system plays a major role in the elimination of inhaled particles by subsequent ingestion into the gastrointestinal system and excretion through the bowels [37, 38]. Muco-ciliary clearance is the primary innate defense mechanism of the lungs, and it involves the mucous layer, airway surface liquid layer, and cilia. These cilia can propel the particles trapped in the mucous layer after inhalation. At the pharynx, these particles mix with salivary secretions and enter the gastrointestinal tract due to swallowing [31, 37]. Following this mechanism, it was evident that nPS and ^64^Cu were accumulated in the liver by fast clearance from lungs as they might have possibly crossed ABB into the systemic circulation where mPS being larger in size retained in the lungs over longer time interval following muco-ciliary clearance as evident from the increased stomach uptake in mPS post-instillation.

The amount of ^64^Cu-DOTA-mPS retained within the lung was significantly higher than that of ^64^Cu-DOTA-nPS. Therefore, mPS might possibly induce higher toxicity in the lung. However, the amount of ^64^Cu-DOTA-nPS retained was significantly higher than that of ^64^Cu-DOTA-mPS in other organs such as spleen, liver, small and large intestine, thymus, kidney, brain, salivary gland, ovary, and urinary bladder, indicating that longitudinal and chronic exposure of nPS may induce more hazardous effects to these organs compared to mPS.

Limitation of this study was spillover effect on PET due to the limited spatial resolution. Although spillover effect was contributed, SUV_max_ and %ID/g values showed a similar pattern or trend.

Taken together, our results demonstrated the utility of PET for visualizing the absorption and distribution of mPS and nPS radiolabeled with ^64^Cu. PET provides information on the accumulation of mPS and nPS in vivo and can provide information on how each organ could be affected following continuous mPS and nPS inhalation. The biological effects of long-term exposure to mPS and nPS in each organ affected in this study will be evaluated in future studies.

## Conclusion

We traced inhaled mPS and/or nPS using ^64^Cu labeling technique and PET imaging. We showed that the accumulated amount of mPS or nPS between lungs and other interesting organs were different depending on the size of PS. These findings would be the basis for future toxicity studies on each organ in the future, which requires longitudinal evaluations of toxicity.

### Supplementary Information

**Additional file 1: Fig. S1.** Radiochemical purity and In-vitro stability

**Additional file 2: Fig. S2.** HPLC data and Ex-vivo TLC of organs

**Additional file 3: Fig. S3.** The transverse, coronal and sagittal PET images

**Additional file 4: Fig. S4.** PET representative images

**Additional file 5: Fig. S5.** PET representative images

## Data Availability

For data sharing: please contact kjs@kirams.re.kr.
