# Supplementary material for: Comparison of PET tracing and biodistribution between 64Cu-labeled micro-and nano-polystyrene in a murine inhalation model
Source: Part Fibre Toxicol. 2024 Jan 31;21:2. doi: 10.1186/s12989-023-00561-7 (PMC10829228; doi:10.1186/s12989-023-00561-7)

Figure S1:

**Fig. S1**. (A) Radiochemical purity of [^64^Cu]Cu-DOTA-mPS and [^64^Cu]Cu-DOTA-nPS analyzed by instant-thin layer chromatography with 0.1 M citric acid as mobile phase. (B) Radiochemical stability of [^64^Cu]Cu-DOTA-mPS and [^64^Cu]Cu-DOTA-nPS. The relative stability was checked in three media; PBS, mouse serum and simulated lung fluid (n=3) for all the time points mentioned in the study. mPS=micro-polystyrene; nPS=nano-polystyrene.


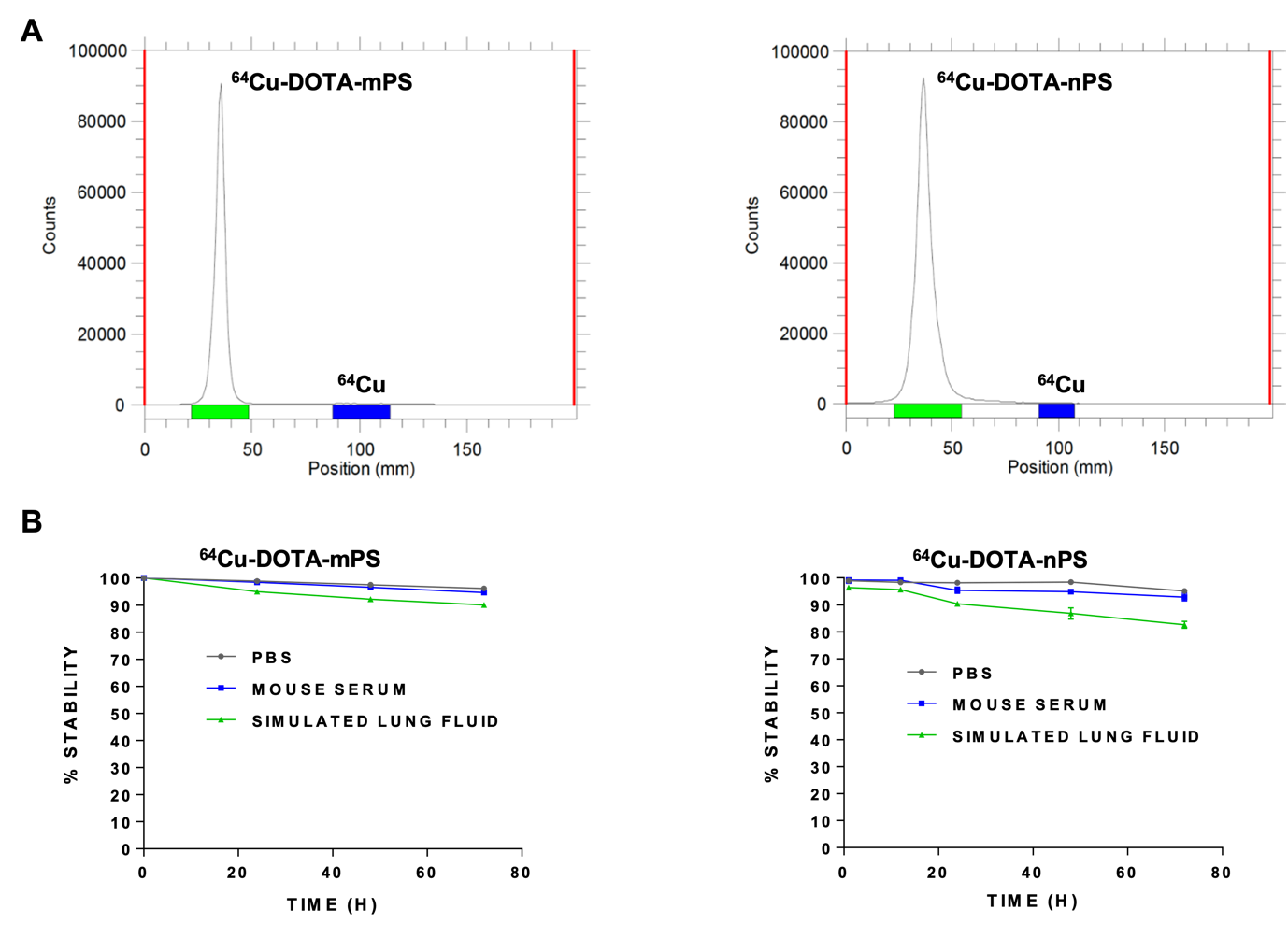

Supplement: Supplementary file 1 — Additional file 1: Fig. S1. Radiochemical purity and In-vitro stability [file 12989_2023_561_MOESM1_ESM.docx]
