# Supplementary material for: Comparison of PET tracing and biodistribution between 64Cu-labeled micro-and nano-polystyrene in a murine inhalation model
Source: Part Fibre Toxicol. 2024 Jan 31;21:2. doi: 10.1186/s12989-023-00561-7 (PMC10829228; doi:10.1186/s12989-023-00561-7)
Supplement: Supplementary file 2 — Additional file 2: Fig. S2. HPLC data and Ex-vivo TLC of organs [file 12989_2023_561_MOESM2_ESM.docx]

Figure S2:


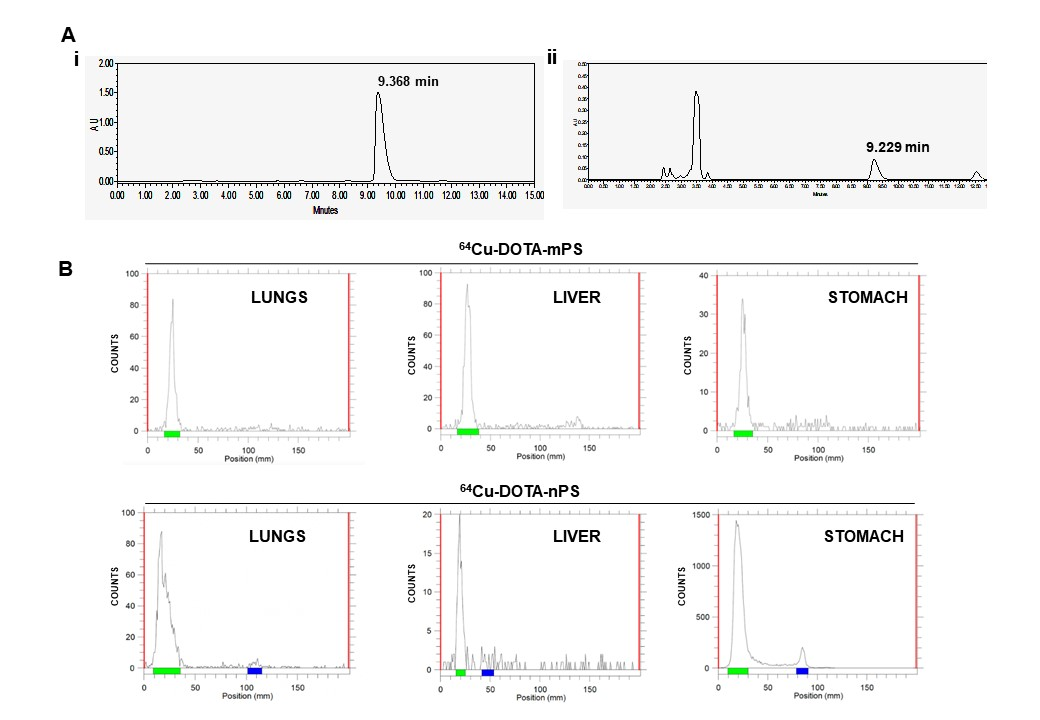


**Fig. S2.** (A) The number of moles of DOTA conjugates per mg of polystyrene was quantified using high performance liquid chromatography; i) HPLC peak of *p*-SCN-Bn-DOTA in the standard concentration of 100 μg/ 50 μl at 254 nm detected at 9.368 min and ii) detection of *p*-SCN-Bn-DOTA in DOTA-polystyrene conjugated product at 9.229 min. (B) *Ex vivo* instant thin layer chromatography results of lungs, liver and stomach at 1 h post intra-tracheal instillation of [^64^Cu]Cu-DOTA-mPS (above) and [^64^Cu]Cu-DOTA-nPS (below) developed from the homogenized tissue samples with 0.1 M citric acid as mobile phase (left peak denotes the [^64^Cu]Cu-DOTA-polystyrene and the right peak is copper-64).
