# Supplementary material for: Comparison of PET tracing and biodistribution between 64Cu-labeled micro-and nano-polystyrene in a murine inhalation model
Source: Part Fibre Toxicol. 2024 Jan 31;21:2. doi: 10.1186/s12989-023-00561-7 (PMC10829228; doi:10.1186/s12989-023-00561-7)
Supplement: Supplementary file 3 — Additional file 3: Fig. S3. The transverse, coronal and sagittal PET images [file 12989_2023_561_MOESM3_ESM.docx]

Figure S3:


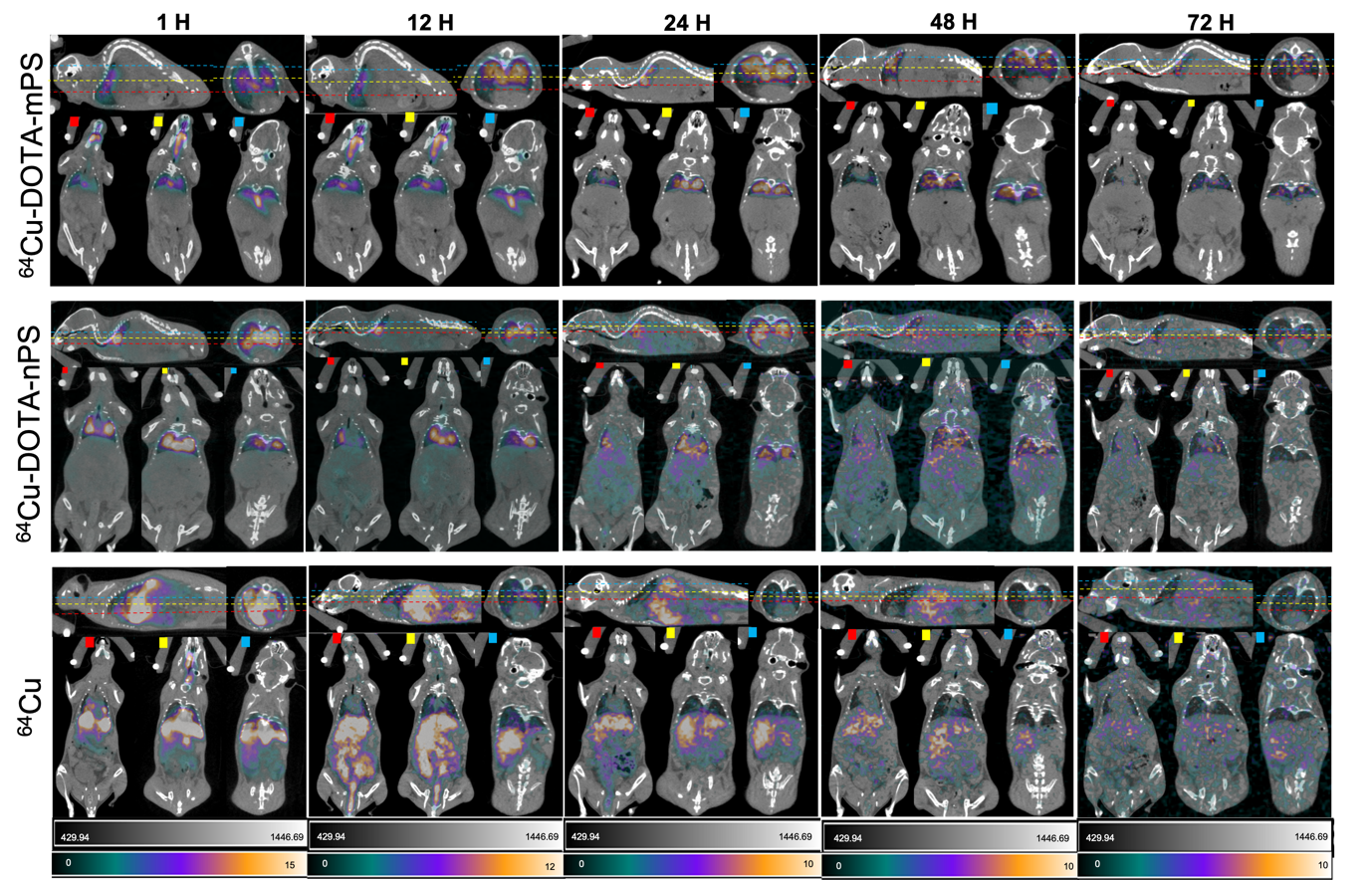


**Fig. S3**. The transverse, coronal and sagittal PET image slices at all time points post instillation. The red, yellow and blue lines indicate anterior, medial and posterior slices respectively and the same slices are represented at different time points.
