# Supplementary material for: Comparison of PET tracing and biodistribution between 64Cu-labeled micro-and nano-polystyrene in a murine inhalation model
Source: Part Fibre Toxicol. 2024 Jan 31;21:2. doi: 10.1186/s12989-023-00561-7 (PMC10829228; doi:10.1186/s12989-023-00561-7)
Supplement: Supplementary file 4 — Additional file 4: Fig. S4. PET representative images [file 12989_2023_561_MOESM4_ESM.docx]

Figure S4:


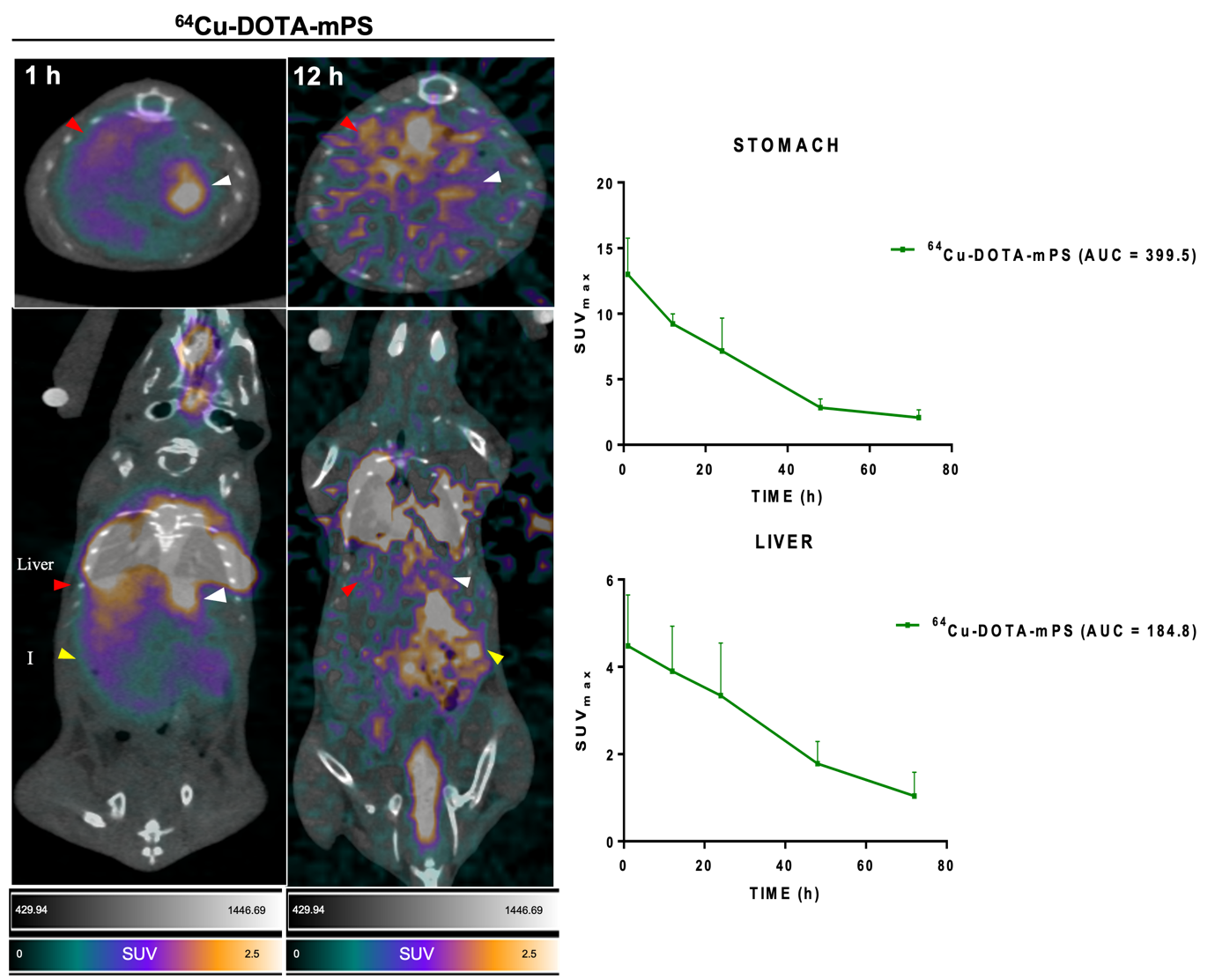


**Fig. S4.** The [^64^Cu]Cu-DOTA-mPS PET images showing stomach and liver uptake. The 1 h and 12 h images showed increased stomach uptake (GIT translocation) and liver uptake in mPS group. The images were marked with arrows pointing to organs; red arrow-liver, white arrow-stomach, yellow arrow-intestines. SUV graph was obtained from Fig. 3.
