# Supplementary material for: Comparison of PET tracing and biodistribution between 64Cu-labeled micro-and nano-polystyrene in a murine inhalation model
Source: Part Fibre Toxicol. 2024 Jan 31;21:2. doi: 10.1186/s12989-023-00561-7 (PMC10829228; doi:10.1186/s12989-023-00561-7)
Supplement: Supplementary file 5 — Additional file 5: Fig. S5. PET representative images [file 12989_2023_561_MOESM5_ESM.docx]

Figure S5:


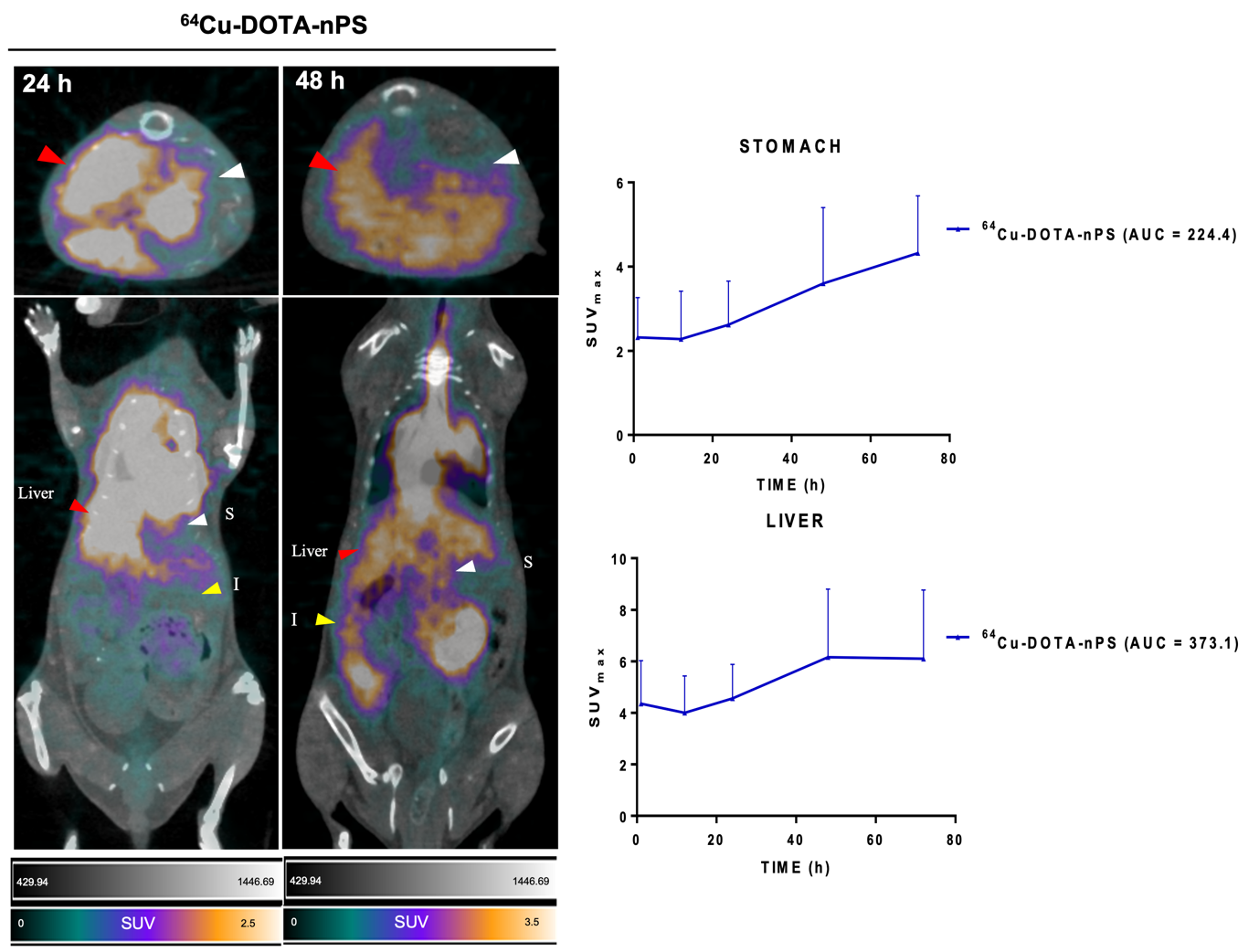


**Fig. S5.** The [^64^Cu]Cu-DOTA-nPS PET images showing stomach and liver uptake. The 24 h and 48 h images showed highest liver uptake in nPS group. The images were marked with arrows pointing to organs; red arrow-liver, white arrow-stomach, yellow arrow-intestines. SUV graph was obtained from Fig. 3.
